# Time to recovery from neonatal sepsis and its determinants among neonates admitted in Woldia comprehensive specialized hospital, Northeast Ethiopia: a retrospective cohort study

**DOI:** 10.3389/fped.2023.1289593

**Published:** 2024-01-25

**Authors:** Kassawmar Ambaye, Ali Yimer, Esuyawkal Mislu, Zeru Wendimagegn, Henok Kumsa

**Affiliations:** ^1^ Department of Maternity and Child Care, Woldia Comprehensive Specialized Hospital, Woldia, Ethiopia; ^2^ Department of Public Health, College of Health Sciences, Woldia University, Woldia, Ethiopia; ^3^ Department of Midwifery, College of Health Sciences, Woldia University, Woldia, Ethiopia; ^4^ Department of Internal Medicine, School of Medicine, College of Health Sciences, Woldia University, Woldia, Ethiopia

**Keywords:** neonatal sepsis, determinants, time to recovery, Woldia, Ethiopia

## Abstract

**Background:**

Neonatal sepsis is the most serious problem in neonates. It is the leading cause of neonatal death in developing countries, particularly in sub-Saharan Africa. The Ethiopian 2016 Demographic Health Survey report revealed that a high number of neonatal deaths are associated with neonatal sepsis. However, limited studies are available on exposure and time to recovery inferences in Ethiopia. Therefore, this study aimed to assess the time to recovery from neonatal sepsis and its determinants among neonates admitted to Woldia Comprehensive Specialized Hospital (WCSH), Northeast Ethiopia.

**Methods:**

A retrospective cohort study was conducted, including 351 neonates, using systematic random sampling at WCSH from 7 to 30 March 2023. The data were entered into Epi data version 4.6 and exported to STATA 14 for analysis. Cox regression was used to identify the determinants of time to recovery from neonatal sepsis, and a variable with a *p*-value of less than 0.05, was used to declare significant association at a 95% confidence interval.

**Result:**

Among 351 neonates with sepsis, 276 (78.63%) recovered, and the median time to recovery was 6 days. Induced labor (AHR = 0.54, 95% CI: 0.369, 0.78) and resuscitation at birth (AHR = 0.7, 95% CI: 0.51, 0.974) were significantly associated with the recovery time of neonatal sepsis.

**Conclusions and recommendation:**

The time to recovery from neonatal sepsis is comparable to previous studies' results. The 25th and 75th percentiles were 4 and 8 days, respectively. Health professionals working in the NICU need to pay special attention to neonates born from mothers who had induced labor and those who were resuscitated at birth.

## Background

Neonatal sepsis (NS) refers to a systemic infection that occurs within the first 28 days of life (1). It is classified into two categories: early-onset NS, which occurs within the first 7 days, and late-onset NS, which occurs after 7 days of age (2–4). NS can be caused by bacterial, viral, or fungal pathogens (5). The clinical presentation of NS is often nonspecific and includes symptoms such as feeding difficulties, fever or hypothermia, respiratory distress, grunting, cyanosis, and apnea (6). The prevalence of specific pathogens causing NS varies across different regions, and reports from developing countries commonly indicate the presence of gram-negative organisms (7).

Sepsis is a significant contributor to global mortality, particularly in the neonatal population. Annually, there are approximately 6.3 million neonatal deaths worldwide, with the majority (73%) occurring in developing countries (8). Neonatal sepsis accounts for an estimated 26% of deaths under 5, with sub-Saharan Africa experiencing the highest mortality rates (9). Moreover, the highest incidence of sepsis is also found in neonates, affecting approximately 3 million infants globally (equivalent to 22 per 1,000 live births). The mortality rate associated with neonatal sepsis ranges from 11% to 19%, and there are also unquantified long-term neurological complications (10).

A study conducted across 12 clinical sites In middle- and low-income countries revealed that the incidence of clinically suspected sepsis was 166 per 1,000 live births, while laboratory-confirmed sepsis occurred at a rate of 46.9 per 1,000 live births. The overall all-cause mortality rate was 0.83 per 1,000 neonates. Notably, the majority of laboratory-confirmed sepsis cases occurred within the first three days of life, indicating a higher prevalence of early-onset neonatal sepsis (11). In line with these findings, the Ethiopian Demographic Health Survey 2016 reported a neonatal mortality rate of 29 per 1,000 live births. Neonatal sepsis was identified as a significant contributing factor to this high number of deaths (12, 13).

The recovery rate and duration of hospital stay for neonates with sepsis vary across different countries and regions. For instance, a study conducted in Central India reported a recovery rate of 61.8% among neonates with sepsis, with an average hospital stay of 9.7 days for those who survived (14). In Southern Ethiopia, approximately 91.4% of septic neonates were reported to recover, with a mean time to recovery of 12.74 days (15). Another study conducted in Central Gondar, Ethiopia, reported a median time to recovery from neonatal sepsis of 7 days (interquartile range, 5–10 days) (16).

Various factors can affect time to recovery from neonatal sepsis. Studies conducted in Central Gondar, Ethiopia, and Sri Lanka showed that maternal chorioamnionitis, preterm labor, intrapartum fever, and PROM are the major determinants of time to recovery from neonatal sepsis (16, 17). Additionally, a study conducted in Tikur Anbessa Specialized Hospital showed that neonates born to mothers with tract infections and prolonged hospitalization had an increased chance of hospital stay (18).

The achievement of the third Sustainable Development Goal for Child Health, which aims to eliminate preventable deaths of newborns and children under 5 by 2030, heavily relies on a substantial reduction in neonatal mortality caused by infections, particularly in developing countries (19). In Ethiopia, there is limited research available on the exposure and time-to-recovery patterns of neonatal sepsis, highlighting the need for further studies in diverse geographical areas to identify the factors influencing recovery in different populations and settings (16). Therefore, this study aimed to assess the time to recovery from neonatal sepsis and its determinants among neonates admitted to the neonatal intensive care unit of the WCSH in Northeast Ethiopia.

## Methods

### Study design and setting

A retrospective follow-up study was conducted from 7–30 March 2023 at Woldia Comprehensive Specialized Hospital (WCSH). It is located approximately 521 km and 360 km from Addis Ababa and Bahir Dar, respectively (20, 21). There were 10 nurses, two neonatal nurses, two general practitioners, and two pediatricians who provided services for admitted neonates. Annually, 750–1,000 neonates are admitted because of various health problems. Approximately 90% of neonates were admitted from the labor ward and approximately 10% were referred from nearby health institutions (22).

### Source populations

The source populations were neonates within 28 days of diagnosis of neonatal sepsis who had been admitted to the NICU of WCSH.

### Study populations

The study populations were neonates within 28 days of being diagnosed with neonatal sepsis who had been admitted to the NICU of WCSH between January 2021 and December 2022.

### Eligibility criteria

#### Inclusion criteria

Neonates diagnosed with neonatal sepsis and admitted to the NICU of WCSH from January 2021 to December 2022 were included in the study.

### Exclusion criteria

The study excluded neonates who died within 30 min of admission.

### Sample size estimation and sampling techniques

The sample size was calculated using STATA software version 14, a sample size for time-to-event data, considering a 95% confidence interval (CI), alpha (0.05), probability of recovery of 0.8098, a hazard ratio for the determinant factors, percentage of recovery, and power of 80% (0.80). We used three variables to estimate the sample size. The sample sizes for the three variables, namely birth weight, intrapartum fever, and time of infection onset, were 351, 313, and 121, respectively, at the Central Gondar Public Hospital (16). Thus, the final sample size was the largest among the 351 mother–newborn pairs.

A systematic random sampling technique was used to recruit the study participants. Using medical numbers of neonates admitted to NICU from January 2021 to December 2022 with neonatal sepsis, a sampling frame was prepared separately for each year. The final sample size was proportionally allocated to each year (124 and 227 neonates in 2021 and 2022, respectively). Lastly, a systematic random sampling technique was used (*K* = *N*/*n* = 774/351 = 2.205 ≈ 2) to select the study participants from the sampling frame.

### Study variables and measurements

The dependent variable was the time to recovery from neonatal sepsis, defined as the time from admission to discharge when the neonate had recovered from NS (1, 15, 16, 23, 24).

**Socio-demographic variables**: maternal age, residence, age of neonate at admission, and neonate sex.

**Maternal-related variables:** parity, gravidity, onset of labor, duration of labor, mode of delivery, place of delivery, ANC visits, multiple pregnancies, Pregnancy-Induced Hypertension (PIH), antepartum hemorrhage, intrapartum fever, duration of PROM, maternal infection history, and chronic illness.

**Neonate-related variables:** birth weight, gestation of neonate at birth, admission weight, temperature, 1-minute and 5-minute Apgar scores, congenital anomalies, resuscitation at birth, meconium aspiration syndrome, respiratory distress, and kept in kangaroo mother care within one hour of birth.

**Clinical & medical care-related and investigation variables:** jaundice, cyanosis, and white blood cell count in the complete blood profile, onset of infection, enteral feeding, critical conditions, and outcome status.

**Healthcare service-related variables:** the prompt initiation of treatment and the timing of seeking medical care after the neonate fell ill.

**Recovery:** if a neonate recovered from the infection after completing treatment, according to the physician's diagnosis.

**Median time to recovery:** the average duration it took for neonates to be declared as recovered by the attending physicians in the unit.

### Operational and term definitions

**Neonatal Sepsis:** if the neonate was diagnosed as having neonatal sepsis by the attending physician after taking a detailed medical history, physical examination, and laboratory tests based on the integrated management of neonatal and childhood illness criteria. The criteria include the presence of two or more persistent fevers (≥37.5°C) or persistent hypothermia (≤35.5°C) lasting for more than 1 h, rapid breathing (≥60 breaths per minute), severe chest indrawing, grunting, poor feeding, movement only when stimulated, a bulged fontanelle, convulsions, lethargy, or unconsciousness. In addition, two or more of the following hematological criteria were used: total leukocyte count (<4,000 or >12,000 cells/mm^3^), absolute neutrophil count (<1,500 cells/mm^3^ or >7,500 cells/mm^3^), platelet count (<150 or >450 cells/mm^3^), and random blood sugar (<40 mg/dl or >125 mg/dl) (4, 25, 26).

**Defaulter**: A neonate is considered a defaulter when they voluntarily stop or leave the treatment unit against medical advice or without completing the prescribed treatment.

**Congenital anomalies**: Congenital anomalies refer to structural or functional abnormalities that are present in a neonate at birth. These anomalies can involve various body systems, such as heart defects, neural tube defects, and genetic conditions like Down syndrome.

**Death**: In the context of this study, death refers to the unfortunate event of a neonate passing away either during the treatment period or while still admitted to the treatment unit due to neonatal sepsis.

**Censored**: A neonate is considered censored when they either default from the treatment, experience death, or are transferred out of the treatment unit. Censored cases are no longer actively followed up or included in the analysis of outcomes.

**Length of stay**: The length of stay refers to the duration in days that a neonate remains in the hospital from the time of admission until the occurrence of an event of interest (such as recovery) or until the neonate is censored (due to defaulter status, death, or transfer out).

**Early initiation of treatment:** Initiate broad-spectrum antimicrobials within the first hour of diagnosis.

**Prolonged rupture of membrane**: The time from membrane rupture to delivery >18 h (27).

**Normal WBC range:** the normal range of WBC is from 5,000–12,000 cells/µl.

**Early initiation of treatment**: initiate broad-spectrum antimicrobials within the first hour of diagnosis.

**Supportive care**: giving care or support with oxygen and feeding without drug management.

**Critical conditions**: when a neonate is hypoxic, hypoglycemic, or unable to maintain a normal body temperature.

### Data collection instruments and procedures

The data for this study were collected using a checklist that was developed based on previous studies. To gather the necessary information, the medical records of both the mothers and neonates were thoroughly reviewed. The checklist encompassed various domains, including neonatal and maternal socio-demographic characteristics, maternal health-related factors, neonatal health-related factors, healthcare service-related factors, and clinical and medical care-related factors. These domains were carefully selected to provide a comprehensive understanding of the factors that may influence the outcomes of neonates with sepsis.

### Data quality assurance

Prior to commencing the data collection process, a preliminary review was conducted on a subset of the sample, specifically 15 neonates, which accounted for 5% of the total sample size. This review served as a pilot study to ensure the effectiveness and feasibility of the data collection tool. The data collection tool was prepared in English, considering the language proficiency of the nursing professionals involved in the study. These professionals were recruited specifically for the purpose of extracting variables from the medical records of the neonate and mother. To ensure the accuracy and consistency of data collection, the investigators provided a comprehensive one-day training session to the nursing professionals. This training covered the purpose and objectives of the study, the techniques for data collection, and the ethical considerations involved in handling patient information.

Throughout the data collection period, the investigators closely monitored the completeness and consistency of the collected data. Daily checks were conducted to identify any discrepancies or missing information, and necessary corrections were made promptly. By conducting a preliminary review, providing appropriate training, and implementing rigorous quality control measures, the study aimed to maintain the integrity and reliability of the collected data.

### Data processing and analysis

After the data collection process, the collected data underwent a series of cleaning, coding, and entry procedures using Epi Data version 4.6. To determine the factors associated with recovery time from neonatal sepsis, Cox regression analysis was conducted. This statistical method allows for the examination of time-to-event data and was deemed appropriate for this study. Initially, variables with a *p*-value less than 0.25 were selected as candidates for the subsequent multivariate Cox regression analysis. In the multivariate analysis, variables with a *p*-value less than 0.05 were considered statistically significant, and their results were reported with 95% confidence intervals. This approach ensured that only the most relevant and impactful factors were considered in determining recovery time. Additionally, to address any potential issue of multicollinearity, the time required for recovery was taken into account during the analysis. Finally, the data were presented in figure, table, and narrative form.

## Result

### Socio-demographic characteristics of study participants

A total of 124 out of 273 neonates and 227 out of 501 admitted neonates in the years 2021 and 2022, respectively, were included in the study (a total of 351 neonates diagnosed with sepsis). The majority of the mothers of these neonates were aged 25–29 (31.91%), followed by those aged 20–24 (29.06%). The mean age of the neonates upon admission was 42 h (1.75 days), and the mean age of the mothers was 26.9 years. Out of the total number of neonates in the study, 270 (76.29%) were only one day old, and 221 (62.96%) were male. More than half of the respondents were living in rural areas (52.71%) (Table 1).

**Table 1:** Socio-demographic characteristics of neonates admitted with neonatal sepsis in WCSH, Northeast Ethiopia, 2022 (*n* = 351).

Variable	Categories	Frequency	Percentage (%)
Age of mother	15–19	34	9.69
	20–24	102	29.06
	25–29	112	31.91
	30–34	61	17.38
	35–49	42	11.97
Residence	Urban	166	47.29
	Rural	185	52.71
Neonatal age on admission	Within 1day	270	76.92
	1–6 days	47	13.39
	≥7 days	34	9.69
Sex of neonate	Male	221	62.96
	Female	130	37.04

### Maternal health-related factors of study participants

Of the neonates admitted to the NICU with NS, 299 (85.19%) were delivered at the same facility. More than half of the mothers were primiparous (51.28%), and the majority had ANC visits. Furthermore, 28 (7.98%) mothers had a history of infection during their pregnancy, and 40 (11.4%) of the mothers gave birth to twins, with either one developing neonatal sepsis. Among the mothers of admitted neonates, 78 (22.22%) had prolonged labor (Table 2).

**Table 2:** Maternal health-related factors of neonates admitted with neonatal sepsis in WCSH, Northeast Ethiopia, 2022 (*n* = 351).

Variable	Categories	Frequency	Percentage (%)
Gravidity	Prim gravida	176	50.14
	Multigravida	175	49.86
Parity	Primiparous	180	51.28
	Multiparous	171	48.72
ANC follow-up	Yes	285	81.20
	No	66	18.80
Multiple pregnancies	Yes	40	11.40
	No	311	88.60
Onset of labor	Spontaneous	297	84.62
	Induced	54	15.38
Place of delivery	Out of this hospital	52	14.81
	In this hospital	299	85.19
Mode of delivery	SVD	197	56.13
	Instrumental vaginal	64	18.23
	Cesarean section	90	25.64
Duration of labor	Normal	273	77.78
	Prolonged	78	22.22
PROM	Yes	74	21.08
	No	277	78.92
Duration of PROM	Normal	58	78.38
	Prolonged	16	21.62
Maternal fever	Yes	22	6.27
	No	329	93.73
APH	Yes	13	3.70
	No	338	96.30
PIH	Yes	20	5.70
	No	331	94.30
Maternal infection history	Yes	28	7.98
	No	323	92.02
Chronic illness history	Yes	11	3.13
	No	340	96.87

ANC, antenatal care; APH, antepartum hemorrhage; PIH, pregnancy-induced hypertension; PROM, premature rupture of membrane; SVD, spontaneous vaginal delivery.

### Neonatal health-related factors of study participants

Among the neonates admitted with NS, 248 (70.66%) were at 37–42 weeks of gestational age and 134 (38.18%) weighed less than 2.5 kg at admission. In total, 127 (36.18%) of them had a 1-minute Apgar score of seven or above. Approximately 11 neonates with neonatal sepsis (3.13%) had a congenital abnormality (Table 3).

**Table 3:** Neonatal health-related factors of neonates admitted with neonatal sepsis in WCSH, Northeast Ethiopia, 2022 (*n* = 351).

Variable	Categories	Frequency	Percentage (%)
Gestational age at birth	<37 weeks	95	27.07
	37–42 weeks	248	70.66
	>42 weeks	8	2.28
1-minute apgar score	Unknown	17	4.84
	<7	207	58.97
	≥7	127	36.18
5-minute apgar score	Unknown	17	4.84
	<7	137	39.03
	≥7	197	56.13
Birth weight	<2.5 kg	135	38.46
	2.5–4 kg	208	59.26
	>4 kg	8	2.28
Weight at admission	<2.5 kg	134	38.18
	2.5–4 kg	206	58.69
	>4 kg	11	3.13
Admission temperature (⁰C)	<36.5	181	51.57
	36.5–37.5	118	33.62
	>37.5	52	14.81
Admission respiratory rate	<30	30	8.55
	30–60	262	74.64
	>60	59	16.81
EBF initiate within 1st hour	Yes	257	73.22
	No	94	26.78
Kept in KMC within 1st hour	Yes	105	29.91
	No	246	70.09
Resuscitated at birth	Yes	141	40.17
	No	210	59.83
MAS	Yes	34	9.69
	No	317	90.31
RDS	Yes	77	21.94
	No	274	78.06
Congenital anomalies	Yes	11	3.13
	No	340	96.87

EBF, exclusive breastfeeding; KMC, Kangaroo mother care; MAS, meconium aspirated syndrome; RDS, respiratory distress syndrome.

### Clinical and medical investigations and health service-related factors

Among the neonates admitted with sepsis, 317 (90.31%) had early-onset neonatal sepsis and 267 (76.07%) initiated treatment within 1 h. Among those with CBC profiles, 170 (48.43%) were within the normal range of WBC counts (Table 4).

**Table 4:** Clinical and medical investigations and health service-related factors of neonates admitted with neonatal sepsis in WCSH, Northeast Ethiopia, 2022.

Variable	Category	Frequency	Percentage (%)
Severe jaundice	Yes	24	6.84
	No	327	93.16
Cyanosis	Yes	21	5.98
	No	330	94.02
Onset of infection	EONS	317	90.03
	LONS	34	9.97
WBC in CBC profile	Normal	170	48.43
	Abnormal	149	42.45
	No information	32	9.12
Non-oral enteral feeding	Yes	149	42.45
	No	202	57.55
Being in a critical condition	Yes	143	40.74
	No	208	59.26
Antibiotics given	Yes	340	96.87
	No	11	3.13
Supportive care	Yes	177	50.43
	No	174	49.57
Blood transfusion	Yes	28	7.98
	No	323	92.02
Anticonvulsant	Yes	30	8.55
	No	321	91.45
Phototherapy	Yes	23	6.55
	No	328	93.45
Weight at discharge (in grams)	<2,500	115	32.76
	2,500–4,000	230	65.53
	>4,000	6	1.71
Early initiation of treatment	Yes	267	76.07
	No	84	23.93
Time to health facility visit after onset of illness	≤3 h	183	52.14
	>3 h	168	47.86

WBC, white blood count; CBC, complete blood count; EONS, early-onset neonatal sepsis; LONS, late-onset neonatal sepsis.

### Sepsis outcome and time to recovery analysis

**Figure 1:**
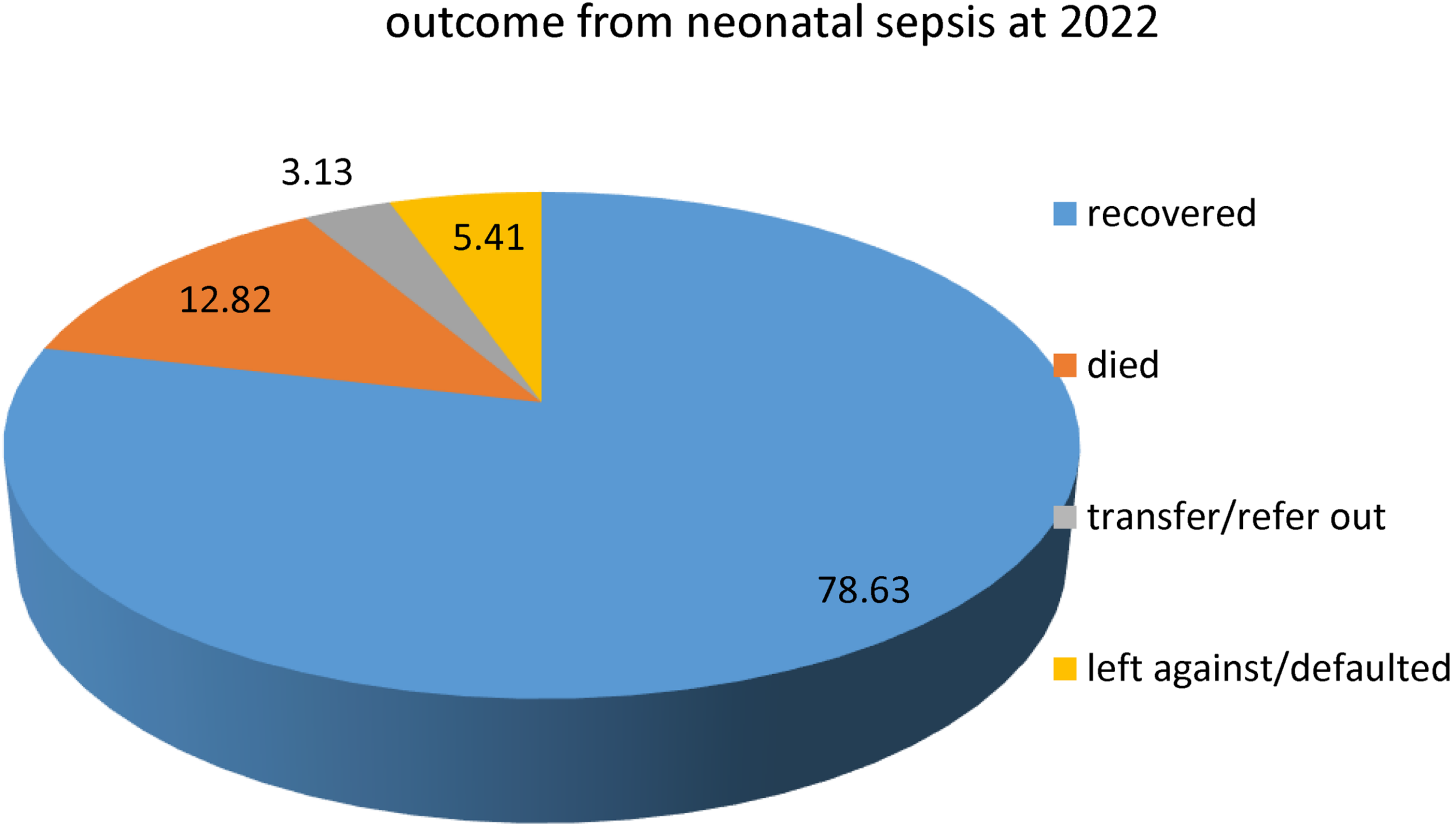
Outcomes of neonatal sepsis for neonates admitted with neonatal sepsis in WCSH, Northeast Ethiopia, 2022.

Among the 351 study participants, 276 (78.63%) successfully recovered from NS, 45 (12.82%) died, 11 (3.13%) were referred, and 19 (5.41%) defaulted/left against medical advice (Figure 1). The mean time to recovery was nearly 7 days, with IQR = 5–10 days, and the median time was 6 days. For preterm neonates included in the study, the mean time to recovery was nearly 8 days (7.71 days) and the median recovery time was 8 days, with IQR from 6 days to 10 days.

**Figure 2:**
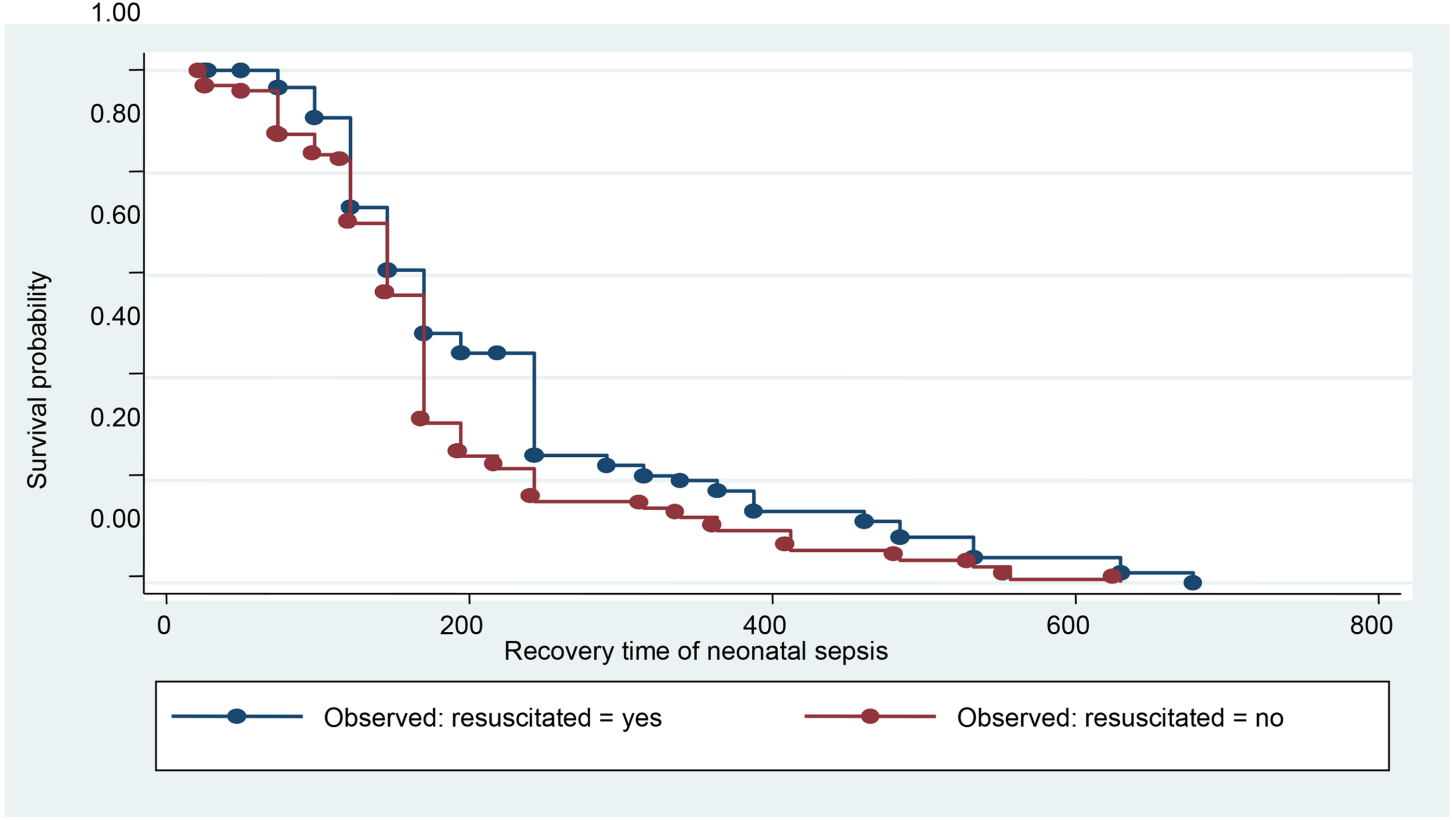
Kaplan–Meier survival curve of neonatal sepsis by resuscitation.

Kaplan–Meier survival proportional hazard graph was used to check the assumption of Cox proportional hazard. The Kaplan–Meier survival curve, performed on the time to recovery in neonates with sepsis based on resuscitation at birth, showed that recovery was delayed among neonates who were resuscitated at birth compared to those who were not resuscitated (Figure 2).

**Figure 3:**
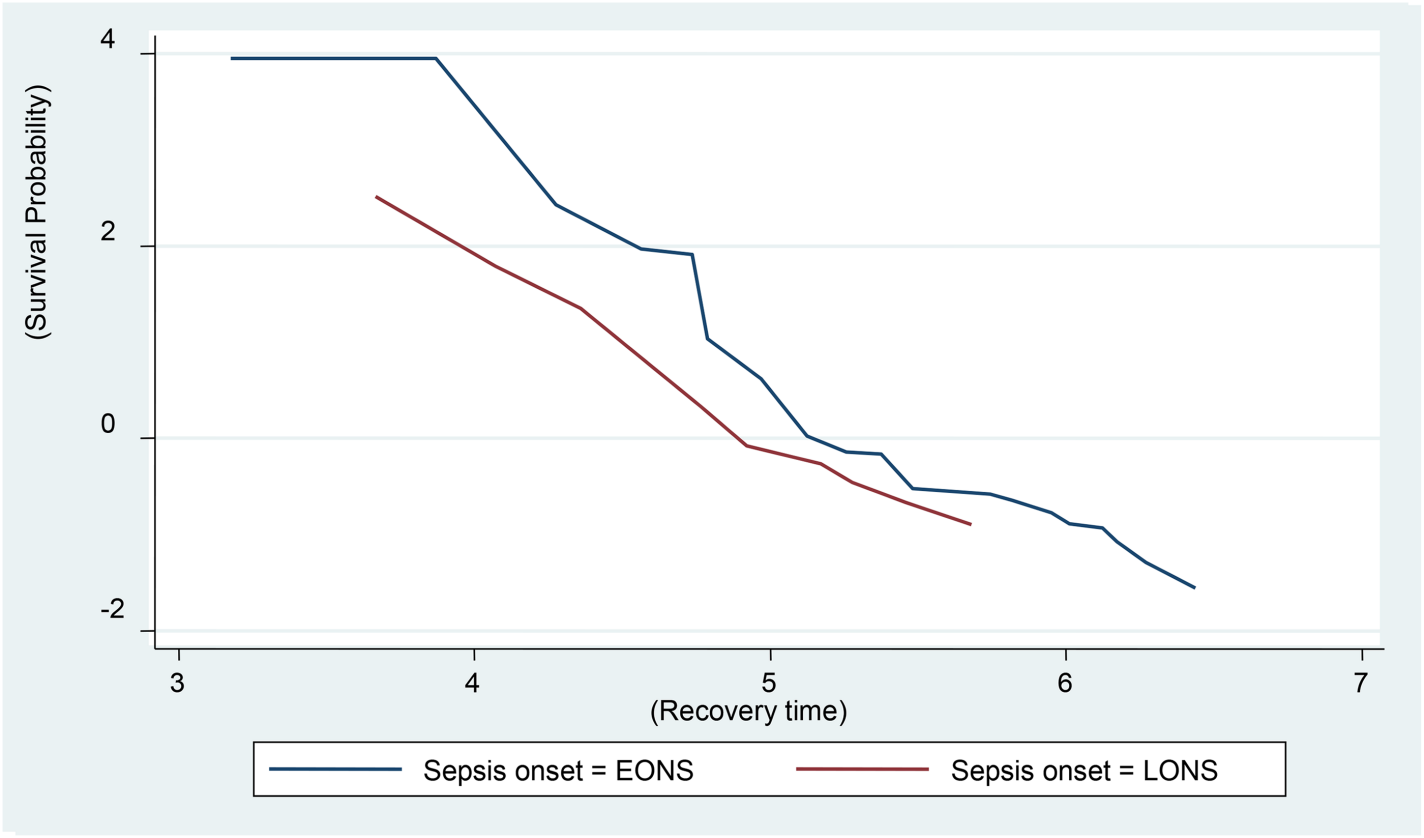
Cox proportional hazards regression survival graph of neonatal sepsis.

The Cox proportional hazards regression survival graph shows the time to recovery of neonates with sepsis, based on the onset of sepsis. Therefore, in neonates with sepsis, EONS has a higher probability of recovery than LONS (Figure 3). In addition, the global test was performed with a *χ*^2^ of 5.92 and a *p*-value of 0.2056, which is greater than 0.05. The dependent variable was std. err of 2.668803 and 95% CI: (170.2511, 180.7489).

### Determinants of time to recovery from neonatal sepsis

After testing, each variable in the study with bivariable analysis (*p* ≤ 0.25) was entered into the multivariable Cox regression analysis (Table 5). Multivariable Cox regression model analysis was performed to show the association between covariates and time to recovery from neonatal sepsis.

**Table 5:** Cox regression analysis showing the association between covariates and time to recovery from neonatal sepsis in WCSH (*n* = 351).

Variable	Recovery from neonatal sepsis		CHR (95% CI)	AHR (95% CI)	*p*-value
	Censored	Event			
Pregnancy					
Multigravida	6	34	0.775 (0.54, 1.11)	0.84 (.545, 1.29)	0.427
Primigravida	69	242	1	1	
Onset of labor					
Spontaneous	65	232	1	1	0.001*
Induced	10	44	.627 (0.45, 0.87)	0.54 (.369, .78)	
Duration of labor					
Normal	47	226	1	1	0.624
Prolonged	28	50	1.26 (.926, 1.72)	1.09 (.77, 1.54)	
APH					
Yes	2	11	0.79 (0.37, 1.26)	0.84 (.429, 1.65)	0.616
No	73	265	1	1	
PIH					
Yes	5	15	1.52 (0.90, 2.56)	1.12 (.61, 2.056)	0.709
No	70	261	1	1	
Gestational age at birth					
<37 weeks	22	73	0.83 (0.63, 1.08)	1.02 (.689, 1.518)	0.909
37–42 weeks	51	197	1	1	
>42 weeks	2	6	0.40 (0.18, 0.91)*	0.46 (.172, 1.259)	0.132
Birth weight					
<2.5 kg	35	98	0.67 (0.52, 0.86)*	0.31 (.089, 1.049)	0.060
2.5–4 kg	340	175	1	1	0.891
>4 kg	1	2	0.95 (0.47, 1.94)	1.07 (.41, 2.78)	
Admission weight					
<2.5 kg	33	99	0.68 (0.53, 0.88)*	2.07 (.577, 7.42)	0.264
2.5–4 kg	42	171	1	1	0.779
>4 kg	1	5	0.68 (0.37, 1.25)	1.13 (.485, 2.625)	
Temperature					
<36.5°C	45	136	1.18 (0.90, 1.54)	1.15 (1.19, 2.06)	0.090
36.5–37.5°C	24	94	1	1	0.858
> 37.5°C	6	46	0.85 (0.59, 1.22)*	1.04 (.69, 1.555)	
RR at admission					
<30	10	20	1.01 (.63, 1.59)	1.46 (.839, 2.537)	0.180
30–60	50	212	1	1	0.081
>60	15	44	0.71 (.51, .994)*	0.72 (.503, 1.04)	
KMC within 1 h					
Yes	17	88	0.80 (0.62, 1.03)	0.93 (.65, 1.329)	0.694
No	58	188	1	1	
Resuscitation at birth					
Yes	34	107	0.76 (0.59, 0.96)	0.70 (.51, .974)	0.034*
No	41	169	1	1	
RDS					
Yes	20	57	0.75 (0.55, 1.02)	0.98 (.655, 1.458)	0.910
No	55	219	1	1	
Initiation of treatment					
Early	63	204	1	1	0.165
Late	12	72	1.43 (1.09, 1.88)	1.27 (.906, 1.779)	
Time of seeking care					
3 or more hours	44	139	1	1	0.214
>3 h	31	137	0.84 (.656, 1.063)	0.83 (.61, 1.117)	
Cyanosis					
Yes	10	11	1.47 (0.80, 2.69)	1.4 (.708, 2.786)	0.331
No	65	265	1	1	
In critical condition					
Yes	50	93	0.79 (0.61, 1.02)	0.96 (.69, 1.338)	0.824
No	25	183	1	1	
IV antibiotics					
Yes	65	275	0.19 (0.023, 1.57)	0.19 (0.02, 1.604)	0.128
No	10	1	1	1	

APH, antepartum hemorrhage; PIH, pregnancy-induced hypertension; RR, respiratory rate; IV, intravenous; KMC, Kangaroo mother care; RDS, respiratory distress syndrome; 1, reference category.

* Significant at *p* < 0.05.

Labor Induction [AHR = 0.538, 95% CI: (0.369, 0.78)] and resuscitation at birth [AHR = 0.7, 95% CI: (0.51, 0.974)] have a significant association with the recovery time of neonates. Cox proportional hazard regression analysis showed that the chance of recovery decreased as the length of hospital stay increased.

## Discussion

This study assessed determinants of time to recovery from neonatal sepsis among neonates admitted to the WCSH in Northeast Ethiopia. Among the 351 neonates with sepsis, 276 (78.63%) recovered, and the median time to recovery was 6 days. Induction of labor and resuscitation at birth have a significant association with recovery time.

This study found that 78.63% of the patients successfully recovered from neonatal sepsis. This is lower than the findings of the study conducted in Dire Dawa (23). This might be due to the higher censoring of admitted septic neonates in the study area of scarce resources because of the northern Ethiopian conflict. Moreover, a greater number of septic neonates admitted were male and rural dwellers, which is in line with studies conducted in Dire Dawa (23) and Central Gondar (16). Furthermore, it was also realized that early-onset neonatal sepsis was the predominant type (90.31%). This finding is congruent with studies conducted in different parts of Ethiopia, such as Mekelle (28), Central Gondar (16), Dire Dawa (23), and Debrezeit (29). A similar finding was also reported in a Western African study conducted in Ghana (30).

The median time to recovery from NS in this study was 6 days. This finding corresponds to the 7-day findings of Dire Dawa (23) and Central Gondar (16). This is also comparable to the findings of earlier studies conducted in Uganda (31) and India (32), which reported median times to recovery for septic neonates of 4.5 and 5.5 days, respectively. These studies shared certain characteristics with the current investigation, such as being conducted on newborns admitted to public hospitals, having a neonate age limit of 0–28 days, having similar sample sizes, and considering clinically confirmed cases.

However, compared to a study conducted in Central India, the mean time to recovery of neonates was 9.67 days (14). This dissimilarity could be attributed to the differences in the study population. Unlike the current study, all neonates included in the Central India study were referred cases or high-risk populations who had a higher likelihood of delayed recovery due to factors such as delay in seeking health care or delay in referral. Additionally, nearly 50% of neonates had low birth weight (LBW) (14), which predisposes them to a lengthy recovery period, whereas our study had a smaller proportion of LBW neonates.

Furthermore, the current study finding was lower than that of the study conducted in the Arba Minch, Sawla, and Chencha hospitals, which indicated that the mean time to recovery of newborns was 12.74 days (15). The observed difference in this study can be attributed to differences in the diagnosis of neonatal sepsis. In the study conducted in Arba Minch, Sawla, and Chencha hospitals, neonatal sepsis was identified by blood culture (15). As a result, the study recovery time may have been longer than the median recovery time in the current study. In addition, the difference in recovery times could be attributed to the difference in the proportion of preterm neonates admitted to the NICU who had a high risk of delayed recovery, at 60% (15) compared to 22.5% in the current study. Furthermore, the proportion of LBW neonates (44%), which was larger than the current study, could also explain this disparity.

The time to recovery from NS is mainly influenced by factors such as induced labor and resuscitation at birth. Neonates delivered from mothers who had induced labor had a 46% delay in time to recovery from NS compared to those born to mothers whose labor started spontaneously. The findings of this investigation are in agreement with studies conducted in Central Gondar (16) and Arba Minch, Sawla, and Chencha hospitals (15). Since women who undergo induction of labor often have certain health problems, the neonate may be initially exposed to risks that could lead to adverse outcomes. Furthermore, it is recommended to offer induction of labor for ruptured membranes, even if it is associated with unfavorable outcomes (15, 33). This could prolong the duration of recovery from NS.

A longer time to recovery from sepsis was observed in neonates who required resuscitation at birth. Comparable findings were revealed in a study done in Central Gondar (16) and in a systematic review of the prognosis (34), indicating that resuscitation with a bag and mask was associated with a 28% delay in recovery from neonatal sepsis. Additionally, studies conducted in Ethiopia (24, 35), Ghana (30), and Tanzania (36) found that resuscitated neonates have a higher chance of developing NS than non-resuscitated neonates. Infants who require resuscitation may face problems in their respiratory or circulatory systems. Furthermore, some resuscitation methods may be invasive for newborns. In addition, neonates who required resuscitation were asphyxiated, and asphyxia lengthened the hospital stay during the NS recovery period.

### Limitations of the study

The diagnosis of NS depended on clinical symptoms, which may result in an increase in false-positive cases, potentially affecting the results of the study.

## Conclusions and recommendations

The time to recovery from sepsis is independently and negatively related to the induction of labor and resuscitation at birth. The final median time to recovery of neonates admitted to the WCSH neonatal intensive care unit was equivalent to that reported in studies conducted in different hospitals in Ethiopia.

Professionals working on NICU wards need to pay special attention to neonates born from mothers who had induced labor and those who were resuscitated at birth. Internal assessment of resuscitation procedures is also important. Further research on confirmed neonatal sepsis cases (EONS and LONS) using prospective follow-up studies in different geographical areas is needed to identify the different determinant factors.

## Data availability statement

The raw data supporting the conclusions of this article will be made available by the authors, without undue reservation.

## Ethics statement

The studies involving humans were approved by Woldia University Institutional review board. The studies were conducted in accordance with the local legislation and institutional requirements. Written informed consent for participation was not required from the participants or the participants’ legal guardians/next of kin in accordance with the national legislation and institutional requirements.

## References

[1] Tewabe T Mohammed S Tilahun Y Melaku B Fenta M Dagnaw T Clinical outcome and risk factors of neonatal sepsis among neonates in Felege Hiwot referral hospital, Bahir Dar, Amhara regional state, North West Ethiopia 2016: a retrospective chart review. BMC Res Notes. (2017) 10(1):1–7. 10.1186/s13104-017-2573-1 28693597 PMC5504561

[2] Assemie MA Alene M Yismaw L Ketema DB Lamore Y Petrucka P Prevalence of neonatal sepsis in Ethiopia: a systematic review and meta-analysis. Int J Pediatr. (2020) 2020:6468492. 10.1155/2020/6468492 32351579 PMC7180396

[3] Sorsa A. Epidemiology of neonatal sepsis and associated factors implicated: observational study at neonatal intensive care unit of Arsi university teaching and referral hospital, South East Ethiopia. Ethiop J Health Sci. (2019) 29(3). 10.4314/ejhs.v29i3.5 31447501 PMC6689722

[4] Ababa A. Neonatal Intensive Care Unit (NICU) Training. Federal Ministry of Health of Ethiopia (2014).

[5] Gebremedhin DBH Gebrekirstos K. Risk factors for neonatal sepsis in public hospitals, of Mekelle City NE, 2015: unmatched case control study. PLoS One. (2016) 11:e0154798. 10.1371/journal.pone.0154798. 27163290 PMC4862626

[6] Elsheshtawy OR Arafa NM Khamis GM. Effect of wee care on physical growth and behavioral responses of preterm neonates. Port Said Sci J Nurs. (2022) 9(2):154–80. 10.21608/PSSJN.2022.94134.1145

[7] Dudeja S. Neonatal sepsis: treatment of neonatal sepsis in multidrug-resistant (MDR) infections: part 2. Indian J Pediatr. (2020) 87(2):122–4. 10.1007/s12098-019-03152-7 31900849

[8] World Health Organization. Neonatal and perinatal mortality: country, regional and global estimates. World Health Organization (2006).

[9] Jayani P Flor M Vectoria A. Neonatal death: case definition gfdc, analysis and presentation of immunization safety data. (2016) 34. 10.1016/j.vaccine.2016.03.040 PMC5139812 27449077

[10] Molloy EJ Wynn JL Bliss J Koenig JM Keij FM McGovern M Neonatal sepsis: need for consensus definition, collaboration and core outcomes. Pediatr Res. (2020) 88:2–4. 10.1038/s41390-020-0850-5 32193517

[11] Milton R Gillespie D Dyer C Taiyari K Carvalho MJ Thomson K Neonatal sepsis and mortality in low-income and middle-income countries from a facility-based birth cohort: an international multisite prospective observational study. Lancet Glob Health. (2022) 10(5):e661–72. 10.1016/S2214-109X(22)00043-2 35427523 PMC9023753

[12] Central SAAA. Ethiopia: Ethiopia Demographic and Health Survey Preliminary Report. In. Measure D, Editor. Calverton, Maryland, USA: ICF., Macro (2011). p. 1–29.

[13] CSA (Ethiopia) and ICF International. Ethiopia Demographic and Health Survey 2016: Key Indicators Report. Addis Ababa and Rockville: CSA (Ethiopia) and ICF International (2016).

[14] Meshram RM Gajimwar VS Bhongade SD. Predictors of mortality in outborns with neonatal sepsis: a prospective observational study. Niger Postgrad Med J. (2019) 26:216–22. 10.4103/npmj.npmj_91_19 31621661

[15] Dessu S Habte A Melis T Gebremedhin M. Survival status and predictors of mortality among newborns admitted with neonatal sepsis at public hospitals in Ethiopia. Int J Pediatr. (2020) 2020:8327028. 10.1155/2020/8327028 33029155 PMC7527886

[16] Oumer M Abebaw D Tazebew A. Time to recovery of neonatal sepsis and determinant factors among neonates admitted in public hospitals of central Gondar zone, northwest Ethiopia, 2021. PLoS One. (2022) 17(7):e0271997. 10.1371/journal.pone.0271997 35900981 PMC9374017

[17] Santhanam S Arun S Rebekah G Ponmudi NJ Chandran J Jose R Perinatal risk factors for neonatal early-onset group B streptococcal sepsis after initiation of risk-based maternal intrapartum antibiotic prophylaxis—a case control study. J Trop Pediatr. (2018) 64(4):312–6. 10.1093/tropej/fmx068 29036682

[18] Sisay EA Mengistu BL Taye WA Fentie AM Yabeyu AB. Length of hospital stay and its predictors among neonatal sepsis patients: a retrospective follow-up study. Int J Gen Med. (2022):8133–42. 10.2147/IJGM.S385829 36389014 PMC9657261

[19] Weiland S Hickmann T Lederer M Marquardt J Schwindenhammer S. The 2030 agenda for sustainable development: transformative change through the sustainable development goals? Politics Gov. (2021) 9(1):90–5. 10.17645/pag.v9i1.4191

[20] Rega S Melese Y Geteneh A Kasew D Eshetu T Biset S. Intestinal parasitic infections among patients who visited Woldia comprehensive specialized hospital’s emergency department over a six-year period, Woldia, Ethiopia: a retrospective study. Infect Drug Resist. (2022):3239–48. 10.2147/IDR.S369827 35761976 PMC9233484

[21] CSA I. Ethiopia Demographic and Health Survey 2011. Addis Ababa, Ethiopia and Calverton, Maryland, USA: Central Statistical Agency and ICF International (2012). p. 430.

[22] Woldia Comprehensive Specialized Hospital. Annual report (2021–2022).

[23] Derese T Belay Y Tariku Z. The median survival recovery time and associated factors among admitted neonate in intensive care units of dire dawa public hospitals, East Ethiopia, 2019. (2020).

[24] Almaw G. Determinants of neonatal sepsis among neonates admitted in neonatal intensive care unit at hospitals of Kafa zone Southwest Ethiopia, 2021: a case control study. (2021).

[25] Ganfure G Lencha B. Sepsis risk factors in neonatal intensive care units of public hospitals in Southeast Ethiopia, 2020: a retrospective unmatched case-control study. Int J Pediatr. (2023) 2023:3088642. 10.1155/2023/3088642 38028728 PMC10657248

[26] Bulto GA Fekene DB Woldeyes BS Debelo BT. Determinants of neonatal sepsis among neonates admitted to public hospitals in central Ethiopia: unmatched case-control study. Glob Pediatr Health. (2021) 8:2333794X211026186. 10.1177/2333794X2110261 34212071 PMC8216335

[27] Akalu TY Gebremichael B Desta KW Aynalem YA Shiferaw WS Alamneh YM. Predictors of neonatal sepsis in public referral hospitals, Northwest Ethiopia: a case control study. PLoS One. (2020) 15(6):e0234472. 10.1371/journal.pone.0234472 32579580 PMC7314009

[28] Gebremedhin D Berhe H Gebrekirstos K. Risk factors, for neonatal sepsis in public hospitals of Mekelle city N, Ethiopia uccs. PLoS One. (2016) 11:e0154798. 10.1371/journal.pone.0154798 27163290 PMC4862626

[29] Woldu MA Guta MB Lenjisa JL Tegegne GT Tesafye G. Assessment of the incidence of neonatal sepsis, its risk factors, antimicrobials use and clinical outcomes in Bishoftu general hospital, neonatal intensive care unit, debrezeit-ethiopia. Pediat Therapeut. (2014) 4:214. 10.4172/2161-0665.1000214

[30] Siakwa M Kpikpitse D Mupepi SC Semuatu M. Neonatal sepsis in rural Ghana: a case control study of risk factors in a birth cohort. Int J Res Med Health Sci. (2014) 4(5).

[31] Tumuhamye J Sommerfelt H Bwanga F Ndeezi G Mukunya D Napyo A Neonatal sepsis at Mulago national referral hospital in Uganda: Etiology, antimicrobial resistance, associated factors and case fatality risk. PLoS One. (2020). 15(8):e0237085. 10.1371/journal.pone.0237085 PMC7416959 32776958

[32] Singh P Wadhwa N Lodha R Sommerfelt H Aneja S Natchu UC Predictors of time to recovery in infants with probable serious bacterial infection. PloS One. (2015) 10(4):e0124594. 10.1371/journal.pone.0124594 25909192 PMC4409397

[33] Liang L Kotadia N English L Kissoon N Ansermino JM Kabakyenga J Predictors of mortality in neonates and infants hospitalized with sepsis or serious infections in developing countries: a systematic review. Front Pediatr. (2018) 6:277. 10.3389/fped.2018.00277 30356806 PMC6190846

[34] Mao DH Miao JK Zou X Chen N Yu LC Lai X Risk factors in predicting prognosis of neonatal bacterial meningitis’a systematic review. Front Neurol. (2018) 9:929. 10.3389/fneur.2018.00929 30515129 PMC6255960

[35] Alemu M Ayana M Abiy H Minuye B Alebachew W Endalamaw A. Determinants of neonatal sepsis among neonates in the Northwest part of Ethiopia: case-control study. Ital J Pediatr. (2019) 45(1):1–8. 10.1186/s13052-019-0739-2 31779698 PMC6883598

[36] Jabiri A Wella HL Semiono A Saria A Protas J. Prevalence and factors associated with neonatal sepsis among neonates in Temeke and Mwananyamala hospitals in Dar es Salaam, Tanzania. Tanzania J Health Res. (2016) 18(4). 10.4314/thrb.v18i4.4

